# Regression of CD30‐positive large cell transformation arising on patch lesion of early mycosis fungoides

**DOI:** 10.1002/ccr3.3171

**Published:** 2020-07-22

**Authors:** Naoko Kubo, Munenari Itoh, Yoshinori Watanabe, Yoshimasa Nobeyama, Akihiko Asahina

**Affiliations:** ^1^ Department of Dermatology The Jikei University School of Medicine Tokyo Japan

**Keywords:** CD30‐positive lymphoproliferative disorder, cutaneous T‐cell lymphoma, early stage, large cell transformation, mycosis fungoides

## Abstract

CD30‐positive large cell transformation that occurs in early mycosis fungoides potentially possesses characteristics of spontaneous regression as with CD30‐positive lymphoproliferative disorders. Such transformation may not relate to poor prognosis.

## Graphical abstract

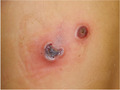

## 1 INTRODUCTION

We present the case of a patient with patch‐stage mycosis fungoides who developed ulcerative tumors compatible with large cell transformation. Tumor cells were positive for CD30 and CXCR3 and negative for CCR3, and subsequently exhibited spontaneous regression. Thus, large cell transformation in early mycosis fungoides may not indicate poor prognosis.

Mycosis fungoides (MF) is the most common type of cutaneous T‐cell lymphoma. Although most MF cases show indolent clinical courses, some cases exhibit severe progression. Previous studies have demonstrated that the prognosis for patients with MF depends on several parameters, including age, clinical stage, and specific factors such as large cell transformation (LCT).^1^, ^2^, ^3^, ^4^, ^5^, ^6^ LCT is diagnosed based on large cells (whether CD30 positive or negative) constituting >25% of the infiltrate or forming microscopic nodules within the MF lesion. LCT of MF (MF‐LCT) has been observed in 2.3%‐22.6% of MF patients during the course of the disease.^2^, ^3^, ^4^, ^5^, ^6^, ^7^, ^8^, ^9^, ^10^, ^11^ Notably, previous studies reported the development of LCT in 21.3%‐31.0% of advanced‐stage patients, but in only 1.4% of early‐stage patients.^3^, ^7^ Thus, MF‐LCT usually is associated with an advanced stage of the disease.

CD30 is a cell membrane protein of the tumor necrosis factor receptor family. CD30 is expressed in activated T cells and used as a tumor marker. Neoplastic proliferation of CD30+ lymphocytes in the skin is observed in MF‐LCT and CD30+ lymphoproliferative disorders (LPDs) such as lymphomatoid papulosis (LyP) and primary cutaneous anaplastic large cell lymphoma (PC‐ALCL). Most LyP cases and about 20% of PC‐ALCL cases spontaneously regress with excellent prognoses.^12^, ^13^, ^14^ However, spontaneous regression of MF‐LCT has been reported only rarely. Here, we present a case showing spontaneous regression of MF‐LCT in patch‐stage MF.

## 2 CASE REPORT

**Figure 1:**
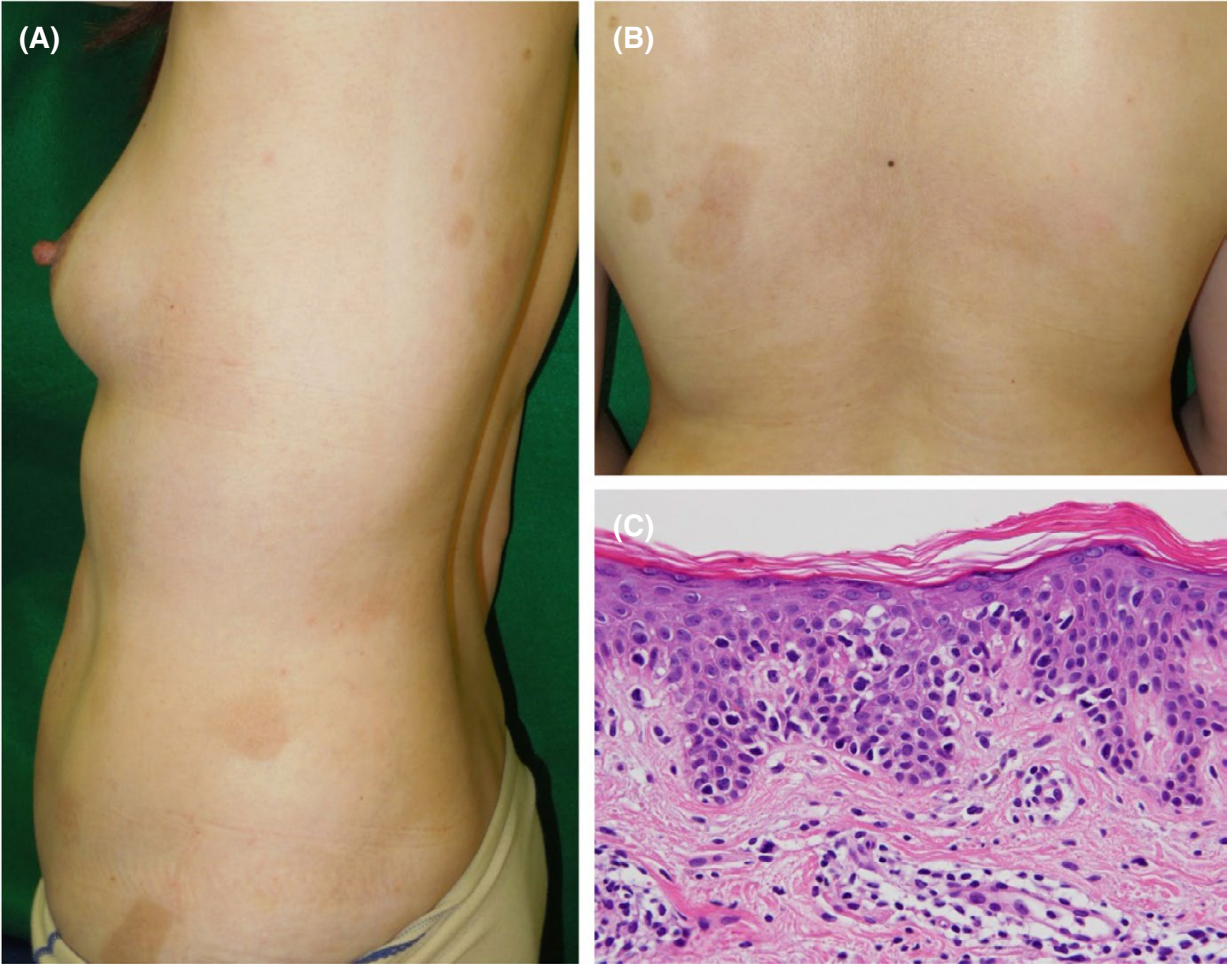
Clinical manifestations at the first visit. A, B, Macroscopic findings. Multiple areas of erythema measuring 1‐10 cm in diameter are evident. C, Histopathological findings of the erythema (hematoxylin‐eosin stain, ×200). Small‐sized atypical lymphocyte‐like hyperchromatic cells with haloes had infiltrated into the epidermis and upper dermis in a scattered manner

A 46‐year‐old Japanese woman was referred to us with a 2‐year history of multiple areas of erythema measuring 1‐10 cm in diameter (Figure 1A, B). The patient had been treated using topical steroids and narrow‐band ultraviolet B irradiation for 2 years in another clinic. Peripheral blood tests showed a normal hemogram and a normal range of soluble interleukin‐2 receptor; the patient was seronegative for anti‐human T‐cell leukemia virus type‐1 antibody. Histopathological examination revealed that small‐sized atypical lymphocyte‐like hyperchromatic cells with haloes had infiltrated into the epidermis and upper dermis in a scattered manner (Figure 1C). Tumor cells were primarily positive for CD4 but negative for CD30. The patient was diagnosed with patch‐stage MF. Symptoms were well controlled by topical steroids and narrow‐band ultraviolet B irradiation therapy.

**Figure 2:**
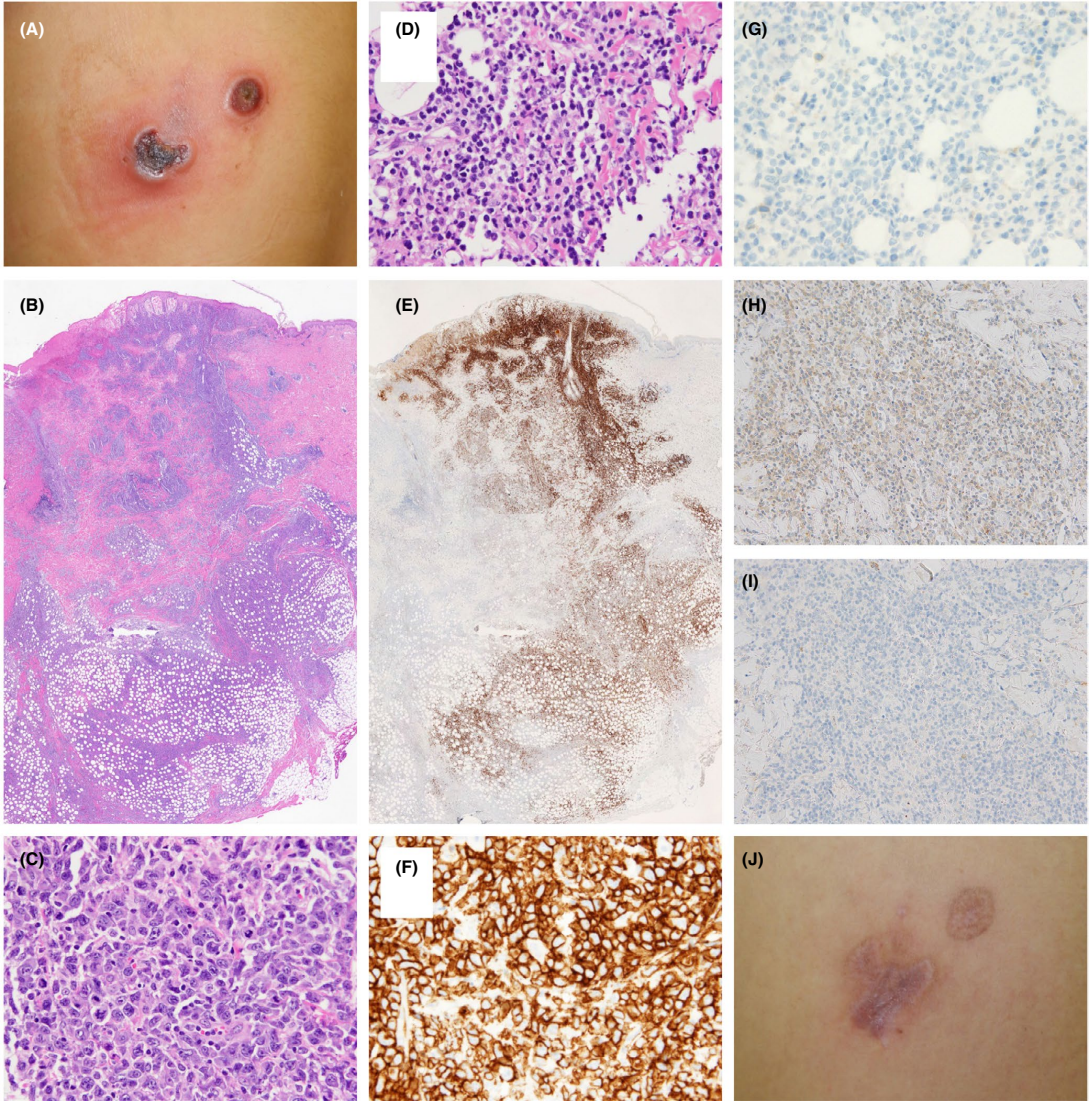
Clinical manifestations during the course of the disease. A, Macroscopic findings at two years after the first visit. Two ulcerative tumors are evident on MF patch lesions of the left chest. B, Histopathological findings of the tumor (hematoxylin‐eosin stain, loupe image). Nodular infiltration of tumor cells is evident in the dermis and subcutis. C, Histopathological findings of the tumor (hematoxylin‐eosin stain, ×400). Some nests consist of anaplastic large cells. D, Histopathological findings of the tumor (hematoxylin‐eosin stain, ×400). Small‐sized atypical lymphocyte‐like hyperchromatic cells have proliferated in the tumor. E, Immunohistochemical examination of the tumor using anti‐CD30 antibody (Roche, Basel, Switzerland) (loupe image). Anti‐CD30 antibodies are reactive to nearly half of the cells in the tumor. F, Immunohistochemical examination using anti‐CD30 antibody (×400). Anti‐CD30 antibodies are reactive to anaplastic large cells. G, Immunohistochemical examination using anti‐CD30 antibody (×400). Anti‐CD30 antibodies are not reactive to small atypical cells. H, Immunohistochemical examination using anti‐CXCR3 antibody (R&D Systems, Minneapolis, MN) (×100). Anti‐CXCR3 antibodies are reactive to anaplastic large cells. I, Immunohistochemical examination using anti‐CCR3 antibody (R&D systems) (×100). Anti‐CCR3 antibodies are not reactive to anaplastic large cells. J, Macroscopic findings at 3 mo after the initial appearance of the tumors. The tumors regressed spontaneously, leaving scars

Two years later, the patient noticed two rapidly growing tumors located on the MF patch lesion in the left chest; the tumors had formed necrotic ulcers measuring 2‐4 cm in diameter (Figure 2A). Peripheral blood tests showed a normal hemogram and normal range of soluble interleukin‐2 receptor. Imaging examinations revealed no invasion into the viscera. Histopathological examination showed nodular infiltration in the dermis and subcutis with necrotic changes in the epidermis (Figure 2B). Anaplastic large cells (≥4 times the size of a small lymphocyte) had formed nodular nests in the dermis and subcutis (Figure 2C). Small‐sized atypical lymphocyte‐like cells also had formed nodular nests, primarily in the subcutis (Figure 2D). Most tumor cells were positive for CD3, CD4, and MIB‐1 (Ki‐67). Anti‐CD30 antibodies were reactive to the anaplastic large cells (Figure 2E, F), which comprised ≤75% of the tumor cells, but not to the small‐sized atypical lymphocyte‐like cells (Figure 2G). The anaplastic large cells were reactive to antibodies against C‐X‐C motif chemokine receptor 3 (CXCR3) (Figure 2H), but not to antibodies against C‐C chemokine receptor type 3 (CCR3) (Figure 2I). Based on these data, a diagnosis of MF‐LCT in the patch stage was made. Three months after their original appearance, the tumors spontaneously regressed, leaving scars (Figure 2J). In the year since, no recurrence has been observed.

## 3 DISCUSSION

The ulcerative tumors occurring in our patient with MF should not be associated with the tumor stage of typical MF. Typical MF progresses slowly from patch stage to tumor stage through plaque stage over several years, but the tumor lesions in our patient occurred suddenly and grew rapidly. Therefore, the present case is incompatible with the tumor stage of MF.

The ulcerative tumors that occurred in our patient are compatible with MF‐LCT rather than CD30 + LPDs such as PC‐ALCL based on the following criteria: (a) LCT develops on pre‐existing MF lesions, and (b) LCT consists of CD30 + large cells, which comprise ≤75% of tumor cells, and small‐sized atypical lymphocyte‐like cells.^15^, ^16^ Recently, several studies have distinguished between PC‐ALCL and LCT as follows: (a) PC‐ALCL cells exhibit strong expression of CCR3 and weak expression of CXCR3, while, in contrast, (b) MF cells exhibit strong expression of CXCR3 and weak expression of CCR3.^17^, ^18^, ^19^ In our case, more than 25% and less than 75% of the tumor lesions located on patch lesions consisted of small‐sized atypical lymphocyte‐like cells and CXCR3‐positive, CCR3‐negative anaplastic large cells, respectively. Based on these findings, the diagnosis of MF‐LCT was confirmed.

Our case shows unique features: LCT occurred in early MF and regressed spontaneously. With regard to LCT in early MF, only two cases (to our knowledge) of CD30 + MF‐LCT in the patch stage have been described previously, with both showing favorable prognoses.^20^, ^21^ These cases, in combination with the case reported here, strongly suggest that CD30 + MF‐LCT in early MF does not indicate poor prognosis, although MF‐LCT occurring in advanced MF has been reported to relate to poor prognosis.^1^, ^6^ With regard to spontaneous regression of MF‐LCT, there have been only a few case reports ^22^, ^23^; notably, PC‐ALCL was not definitely excluded in those instances. CD30 + MF‐LCT, especially in early MF, also may possess the potential for spontaneous regression like that seen for CD30 + LPDs, including LyP and PC‐ALCL. The accumulation of additional such unique cases is expected to contribute to deeper understanding of MF‐LCT.

## CONFLICT OF INTEREST

None declared.

## AUTHOR CONTRIBUTIONS

NK: provided resources. MI: contributed to concentration, validation, and writing. YW: contributed to data curation. YN: performed project administration. AA: provided supervision.

## ETHICAL APPROVAL

The case report was approved by the ethics committee of The Jikei University School of Medicine.
